# Personality Influences the Relationship Between Primary Emotions and Religious/Spiritual Well-Being

**DOI:** 10.3389/fpsyg.2018.00370

**Published:** 2018-03-19

**Authors:** Michaela Hiebler-Ragger, Jürgen Fuchshuber, Heidrun Dröscher, Christian Vajda, Andreas Fink, Human F. Unterrainer

**Affiliations:** ^1^Department of Psychiatry and Psychotherapeutic Medicine, Medical University of Graz, Graz, Austria; ^2^Center for Integrative Addiction Research, Grüner Kreis Society, Vienna, Austria; ^3^Institute of Psychology, University of Graz, Graz, Austria; ^4^Department of Medical Psychology and Psychotherapy, Medical University of Graz, Graz, Austria; ^5^Department of Religious Studies, University of Vienna, Vienna, Austria

**Keywords:** personality, development, primary emotions, psychological well-being, spirituality

## Abstract

The study of human emotions and personality provides valuable insights into the parameters of mental health and well-being. Affective neuroscience proposes that several levels of emotions – ranging from primary ones such as LUST or FEAR up to higher emotions such as spirituality – interact on a neural level. The present study aimed to further explore this theory. Furthermore, we hypothesized that personality – formed by bottom-up primary emotions and cortical top-down regulation – might act as a link between primary emotions and religious/spiritual well-being. A total sample of 167 (78% female) student participants completed the Affective Neuroscience Personality Scale (primary emotions), the Big Five Personality Inventory and the Multidimensional Inventory of Religious/Spiritual Well-Being (higher emotions). Correlation analyses confirmed the link between primary and higher emotions as well as their relation to personality. Further regression analyses indicated that personality dimensions mediate the relationship between primary and higher emotions. A substantial interaction between primary emotions, personality dimensions, and religious/spiritual well-being could be confirmed. From a developmental perspective, cortical top-down regulation might influence religious/spiritual well-being by forming relevant personality dimensions. Hence, CARE as well as Agreeableness seem of special importance. Future studies might focus on implications for clinical groups.

## Introduction

From an evolutionary standpoint, the experience of spirituality and religiousness can be seen as the result of the same advantageous neuronal development responsible for the human tendency to socialize and create communities (Kirkpatrick, 2005). While spirituality has been described most prominently as “the search for significance in ways related to the sacred” (Pargament, 1999, p. 4) and therefore as a very personal and affective construct, religiousness is largely defined by specific rituals, ideologies, and institutions (Pargament, 1999).

Furthermore, spirituality and religiousness have been linked to mental health in several studies (Espíndola and Blay, 2013; Unterrainer et al., 2014; Hiebler-Ragger et al., 2016). However, studying the neurobiology of spiritual and religious experiences and well-being is a complicated task (Perroud, 2009).

### Affective Neuroscience

Affective neuroscience (AN) is an interdisciplinary field exploring the neuronal mechanisms of emotions. It combines neuroscience with the psychological study of personality, emotion, and mood. Moreover, AN emphasizes the importance of primary-process subcortical brain emotion systems: emotional experiences are not seen as an epiphenomenon but are hypothesized to have an evolutionary function for the survival of every mammalian species since they enable mammals to automatically anticipate several survival concerns (Panksepp and Biven, 2012). Panksepp (1998) described a continuity of primal emotional systems in the subcortical-limbic circuits of selected areas in the mammalian brain. When stimulated these interacting systems evoke seven primary emotions: SEEKING, ANGER, PLAY, FEAR, CARE, PANIC-GRIEF, and LUST. The SEEKING system, arising from the ventral tegmental area, encourages foraging, exploration, investigation, curiosity, interest and expectancy. As a result dopamine fires each time the mammal explores its environment (Depue and Collins, 1999). The shut-down of the SEEKING system, which can be caused by any perception of isolation or loss of love, will trigger the PANIC-GRIEF system. This system activates the psychological pain related to loss and social rejection and often coincides with increased amounts of FEAR and ANGER. By contrast, LUST and PLAY promote social joy in the brain; these systems work together with the SEEKING system (Panksepp and Biven, 2012).

Furthermore, these – genetically influenced (Montag et al., 2016) – primary emotions are seen as the foundation and major force for the development of personality viewed in the context of Darwinian “continuity” which presupposes a common origin for human and animal existence (Fouts and Waters, 2001; Bouchard and Loehlin, 2001; Nettle, 2006; Davis and Panksepp, 2011; Panksepp and Watt, 2011). To examine the effect of primary emotions on various personality dimensions the Affective Neuroscience Personality Scale (ANPS) was developed (Davis and Panksepp, 2011).

### Primary Emotions and Personality

Personality dimensions, most prominently described through the Big Five (Extraversion, Neuroticism, Openness, Consciousness, and Agreeableness), are regarded as universal, intraindividually constant, and interindividually different (McCrae and Costa, 1997; Soldz and Vaillant, 1999; Bouchard and Loehlin, 2001) and are also, at least in part, inheritable (Loehlin et al., 1998). From an evolutionary perspective, the Big Five might have had a possibly adaptive value already in prehistoric times (Buss, 1991) with their development representing the combination of gene expression with the influence of environmental factors (Goldberg, 1990). In correspondence to this, differences in personality structure in general (Uher and Asendorpf, 2007) and specifically in Big Five personality factors (King and Figueredo, 1997) can also be observed in big apes. Furthermore, personality dimensions – especially Neuroticism – have also been linked to various aspects of mental health (Khan et al., 2005; O’Leary-Barrett et al., 2015; Huang et al., 2017).

Davis and Panksepp (2011) report substantial connections between primary emotions and the Big Five: They observed positive correlations between SEEKING and Openness, PLAY and Extraversion as well as CARE and Agreeableness while Neuroticism was positively correlated with ANGER, FEAR, and SADNESS. Conscientiousness was the only personality factor with no connection to the primary emotions (Abella et al., 2011; Barrett et al., 2013). By early adulthood, the resulting personality is relatively stable across time when sufficiently matured upper brain regions allow the prefrontal cortex to regulate arising emotions (Costa and McCrae, 1994 for an overview see; McCrae and Costa, 1994).

Therefore, individual differences in primary emotions such as the “evolutionary oldest parts of human personality” (Montag and Panksepp, 2017, p. 2) seem to form human personality from the bottom-up in tandem with cortical top-down regulation (Montag and Panksepp, 2017). In detail, the “the unique personality of a person will be shaped by the strength of both the tonic/phasic emotional bursts from the subcortical levels of the brain, together with one’s ability to hold a tight grip on the emotional activities (abilities in emotion regulation) – hence cortical, top–down regulation” (Montag and Panksepp, 2017, p. 8).

### Spirituality and Well-Being

Within the framework of AN, spirituality has been named as a higher-order affective human attribute and as an important factor in research related to personality and mental health (Davis and Panksepp, 2011). In detail, Davis et al. (2003, p. 60) defined spirituality as “feeling ‘connected’ to humanity and creation as a whole, feeling a sense of ‘oneness’ with creation, striving for inner peace and harmony, relying on spiritual principles, and searching for meaning in life.” However, while several studies have focused on the primary emotions, there is relatively little research on spirituality within the framework of AN – even in the articles of Davis and Panksepp (2011).

Beyond the framework of AN, spirituality has received extensive attention and is generally described as emotional, namely as “a universal experience, not a universal theology” (Vaughan, 1991, p. 116). Consequently, it applies to theistic (e.g., Christianity), the polytheistic (e.g., Hinduism) and non-theistic beliefs (e.g., Buddhism) (Vaughan, 1991). Furthermore, spirituality may be closely connected to emotion regulation, as the relationship a higher power (e.g., God) is thought to meet the criteria of an attachment relationship and would consequently offer similar psychological advantages (Kirkpatrick, 2005).

In addition, suggestions have been made for the expansion of the classic Big Five to include Spirituality as a sixth factor (Piedmont, 1999; MacDonald, 2000; Unterrainer et al., 2010). This is supported by studies reporting positive correlations of Agreeableness, Conscientiousness, and Extraversion with several aspects of religiousness or spirituality (Saroglou, 2010; Unterrainer et al., 2010). Moreover, mystical or transcendental experiences are mostly reported by individuals with higher levels of Extraversion and Openness (Unterrainer et al., 2014). Likewise, a meta-analytic review reported increased Openness as related to a more open or mature religiousness and spirituality as well as to less religious fundamentalism (Saroglou, 2010).

Furthermore, dimensions of religiosity and spirituality have been linked to various concepts of mental health and illness: A good example might be the concept of Spiritual Well-Being (SWB), coined by Ellison and Smith (1991). SWB includes the God-related or transcendent dimension of Religious Well-Being (RWB) as well as the immanent dimension of Existential Well-Being (EWB) that is connected to life satisfaction and purpose without relying on a higher power (Ledbetter et al., 1991). SWB was demonstrated to be positively correlated with various indicators of mental health such as more positive affective states and adequate stress coping strategies in clinical as well as non-clinical samples (Ledbetter et al., 1991; Hiebler-Ragger et al., 2016). In the multifactorial expansion of this concept, the Multidimensional Inventory for Religious/Spiritual Well-Being (MI-RSWB) (Unterrainer et al., 2010), the initially two dimensions of RWB and EWB (Ledbetter et al., 1991) have each been given three sub-dimensions: Hope Immanent, Forgiveness and Experiences of Sense and Meaning for EWB and Hope Transcendent, General Religiosity and Connectedness for RWB. While the sub-dimensions of EWB are not necessarily connected to religion, they are strongly connected to both religiosity and subjective well-being. Therefore, they might mediate the relationship between religiosity and health (Unterrainer et al., 2010). Regarding the sub-dimensions of RWB, General Religiosity has a stronger connection to religious institutions, communities or traditions, while Connectedness has a stronger deinstitutionalized character including the general belief in a higher power. Lastly, Hope Transcendent implies a lower amount of existential fear as well as the hope for a better life after death (Unterrainer et al., 2010).

Accordingly, RSWB was defined by Unterrainer et al. (2011, p. 117) as “the ability to experience and integrate meaning and purpose in existence through the connectedness with self, others or a power greater than oneself.” This multi-facetted construct therefore can be seen as a health-oriented operationalization of spirituality. In line with this, Ellison states that SWB “arises from an underlying state of spiritual health and is an expression of it, much like the color of one’s complexion and pulse rate are expressions of good [physical] health” (Ellison, 1983, p. 332).

### The Present Study

In the present work, and in line with previous research (Davis and Panksepp, 2011; Montag and Panksepp, 2017), we assumed a substantial relationship between primary emotions, personality factors and spirituality: We hypothesized that stronger negative primary emotions (FEAR, ANGER, and SADNESS) should be related to an increased amount of Neuroticism as well as a decreased amount of RSWB. On the other hand, positive primary emotions (SEEKING, CARE, and PLAY) should be related to an increased amount of more favorable personality traits such as Extraversion and Agreeableness. Furthermore, we intended to investigate the extent to which primary emotions predicted religious/spiritual well-being through the mediating influence of personality.

## Materials and Methods

### Sample Description and Procedure

Participants were included in the study if they were German speaking, between 18 and 30 years of age and had either finished an apprenticeship or were students at the University of Graz, Austria. The questionnaires were completed online on the LimeSurvey^®^ platform. Participants were recruited via social media and e-mail distribution lists. Based on the established parameters (Cohen, 1988), we calculated a required sample size of 150 participants. Recruitment status was checked regularly and stopped when the number of participants that had completed all questionnaires exceeded the required sample size. The Ethics Board of the University of Graz, Austria granted ethical approval and informed consent was obtained from all participants. All the data needed to reproduce the results, as well as the data analysis scripts, are open and can be accessed by contacting the corresponding author.

### Psychometric Assessment

#### Primary Emotions

The *Affective Neuroscience Personality Scales* (ANPS; German version) (Davis and Panksepp, 2011), measures the six neural-based brain networks PLAY, SEEK, CARE, FEAR, ANGER, and SADNESS. Each sub-scale is comprised of 14 items. The additional filler-items are not used in the evaluation of the questionnaire. The items have to be rated on a 4-point Likert scale ranging from 1 (strongly disagree) to 4 (strongly agree).

#### Personality Traits

The *Big Five Inventory* (BFI-44; German version) (John and Srivastava, 1999; Lang et al., 2001) is a 44-item inventory for the assessment of the Big Five factors of personality: Extraversion, Agreeableness, Conscientiousness, Neuroticism, and Openness. Each of the five sub-scales comprises between 8 and 10 items. The items have to be rated on a 5-point Likert scale ranging from 1 (strongly disagree) to 5 (strongly agree).

#### Religious/Spiritual Well-Being

The *Multidimensional Inventory for Religious/Spiritual Well-being* (MI-RSWB) (Unterrainer et al., 2010) contains six sub-dimensions (eight items per sub-dimension): Hope Immanent, Forgiveness and Experience of Sense and Meaning form the dimension Existential Well-Being (EWB), while Hope Transcendent, General Religiosity and Connectedness form the dimension Religious Well-Being (RWB). Each item is rated on a 6-point Likert scale ranging from 1 (strongly disagree) to 6 (strongly agree). By summing up EWB and RWB the total amount of Religious/Spiritual Well-Being (RSWB) can be calculated. For this study only the dimensions EWB and RWB as well as the RSWB total score, were used.

### Statistical Analysis

To investigate the relationship between the study variables Pearson correlation statistics were conducted. In order to test the hypothesized influence of personality on the relationship between primary and higher emotions linear hierarchical regression analyses was conducted as this method allows a theory driven selection of predictors (Lewis, 2007). Furthermore, while more complex procedures for mediation analysis with multiple mediators, e.g., (VanderWeele and Vansteelandt, 2014), would be needed to confirm a hypothesized mediation, hierarchical regression clearly demonstrates how the importance of predictors change when additional predictors are entered and whether those additional predictors add to the variability explained by the model. To avoid alpha-inflation the alpha-level was set to 0.01.

## Results

### Sample Characteristics

A total sample of 167 participants (78% female) was included in the analyses. The mean age of the participants was 23 years (*SD* = 3.33). Most participants were Christian (*n* = 116; 70%), 48 (28%) were without confession, 3 (2%) made no specification. 78 (47%) participants described themselves as religious. 161 (96%) participants were Austrian, 6 (4%) had other nationalities. For highest completed education level, 2 (1%) participants had finished an apprenticeship, 113 (68%) had a high school diploma and 52 (31%) had already completed an undergraduate degree.

### Hypothesis Testing Results

The results of the Pearson correlation statistics, as well as descriptive characteristics of all variables (including Cronbach Alpha), are presented in **Table 1**. As hypothesized, we found that every personality dimension was related to several primary emotions: In particular, associations were found for Extraversion with PLAY (*r* = 0.52, *p* < 0.01), Agreeableness with CARE (*r* = 0.50, *p* < 0.001), Conscientiousness with SEEKING (*r* = 0.34, *p* < 0.001), Neuroticism with FEAR (*r* = 0.76, *p* < 0.001) and Openness with SEEKING (*r* = 0.56, *p* < 0.001).

**Table 1:** Descriptive characteristics and intercorrelations of study variables.

Measures	α	*M*	*SD*	1	2	3	4	5	6	7	8	9	10	11	12	13	14
**ANPS**																	
(1) SEEKING	0.76	41.17	5.53	-													
(2) FEAR	0.86	38.23	7.15	–0.27^***^	–												
(3) CARE	0.84	42.55	6.88	0.22^**^	0.08	–											
(4) ANGER	0.84	36.11	7.04	–0.07	0.37^***^	–0.05	–										
(5) SADNESS	0.75	35.25	5.80	–0.30^***^	0.67^***^	0.13	0.40^***^	–									
(6) PLAY	0.84	42.44	6.81	0.33^***^	–0.30^***^	0.49^***^	–0.01	–0.17	–								
**BFI**																	
(7) E	0.90	3.38	0.82	0.36^***^	–0.33^***^	0.39^***^	–0.00	–0.24^**^	0.52^**^	–							
(8) A	0.70	3.65	0.57	0.05	–0.09	0.50^***^	–0.39^***^	–0.08	0.35^**^	0.30^**^	–						
(9) C	0.85	3.54	0.70	0.34^***^	–0.21^**^	0.13	–0.19	–0.27^**^	0.09	0.27^**^	0.19	–					
(10) N	0.85	3.00	0.76	–0.22^**^	0.76^***^	0.09	0.47^***^	0.65^**^	–0.34^**^	–0.30^**^	–0.20	–0.11	–				
(11) O	0.84	3.80	0.67	0.56^***^	0.00	0.24^**^	0.04	–0.01	0.24^**^	0.22^**^	0.07	0.14	0.00	–			
**MI-RSWB**																	
(12) EWB	0.88	109.04	16.41	0.41^***^	–0.38^***^	0.41^***^	–0.38^***^	–0.32^**^	0.47^**^	0.60^**^	0.54^**^	0.41^**^	–0.38^∗∗^	0.27^∗∗^	–		
(13) RWB	0.88	76.14	19.17	0.19	–0.26^**^	0.32^***^	–0.22^**^	–0.17	0.22^**^	0.35^**^	0.42^**^	0.17	–0.20^∗∗^	0.24^∗∗^	0.60^∗∗^	–	
(14) RSWB	0.92	185.18	31.84	0.32^***^	–0.36^***^	0.40^***^	–0.33^***^	–0.30^**^	0.38^**^	0.52^**^	0.59^**^	0.32^**^	–0.32^∗∗^	0.28^∗∗^	0.88^∗∗^	0.91^∗∗^	–

*^∗∗^p < 0.01, ^∗∗∗^p < 0.001; ANPS, Affective Neuroscience Personality Scales; BFI, Big Five Inventory; E, Extraversion; A, Agreeableness; C, Conscientiousness; N, Neuroticism; O, Openness; MI-RSWB, Multidimensional Inventory of Religious/Spiritual Well-Being; EWB, Existential Well-Being; RWB, Religious Well-Being; RSWB, Religious/Spiritual Well-Being.*

Moreover, all primary emotions and all personality dimensions were related to RSWB and EWB. In particular, relations were found for RSWB with CARE (*r* = 0.40, *p* < 0.001) and Agreeableness (*r* = 0.59, *p* < 0.01) and for EWB with PLAY (*r* = 0.47, *p* < 0.01) and Extraversion (*r* = 0.60, *p* < 0.01). RWB showed the strongest links with CARE (*r* = 0.32, *p* < 0.001) and Agreeableness (*r* = 0.42, *p* < 0.01).

In the hierarchical regression analyses (see **Table 2**), sex was entered as a control variable at Step 1 as we found a trend toward higher levels of RSWB in female compared to male participants [*M*_female_ = 187.87, *M*_male_ = 175.73; *F*_(1,165)_ = 4.27, *p* = 0.04].

**Table 2:** Hierarchical regression analyses predicting Religious/Spiritual Well-Being.

		EWB			RWB			RSWB		
Step and predictor variable		*R*^2^	Δ*R*^2^	β	*R*^2^	Δ*R*^2^	β	*R*^2^	*ΔR*^2^	β
Step 1		0.04	0.04		0.01	0.01		0.03	0.03	
	Sex			–0.19			–0.10			–0.16
Step 2		0.49^∗∗∗^	0.45^∗∗∗^		0.20^∗∗∗^	0.19^∗∗∗^		0.38^∗∗∗^	0.35^∗∗∗^	
	Sex			–0.17^**^			–0.05			–0.12
	SEEKING			0.21^**^			0.06			0.14
	FEAR			–0.16			–0.26^+^			–0.24^+^
	CARE			0.20^**^			0.33^***^			0.30^***^
	ANGER			–0.27^***^			–0.11			–0.21^**^
	SADNESS			–0.07			0.01			–0.03
	PLAY			0.24^**^			–0.04			0.10
Step 3		0.64^∗∗∗^	0.15^∗∗∗^		0.31^∗∗∗^	0.11^∗∗∗^		0.53^∗∗∗^	0.15^∗∗∗^	
	Sex			–0.09			0.02			–0.03
	SEEKING			0.11			–0.05			0.02
	FEAR			–0.09			–0.29^+^			–0.22
	CARE			0.02			0.15			0.10
	ANGER			–0.21^**^			–0.03			–0.13
	SADNESS			0.00			0.01			0.01
	PLAY			0.10			–0.15			–0.04
	Extraversion			0.32^***^			0.18			0.28^***^
	Agreeableness			0.25^***^			0.31^**^			0.31^***^
	Conscientiousness			0.15^**^			–0.01			0.08
	Neuroticism			–0.02			0.07			0.03
	Openness			0.07			0.21			0.16

*^+^p < 0.015, ^∗∗^p < 0.01, ^∗∗∗^p < 0.001; Sex: 1, male; EWB, Existential Well-Being; RWB, Religious Well-Being; RSWB, Religious/Spiritual Well-Being.*

The primary emotions were entered at Step 2, the personality dimensions at Step 3. The hierarchical regression analyses, including all predictors and the control variable, accounted for 64% (adjusted *R*^2^ = 0.61) of the variance in EWB [*F*_(12,154)_ = 22.96, *p* < 0.01], 31% (adjusted *R*^2^ = 0.26) of the variance in RWB [*F*_(12,154)_ = 5.85, *p* < 0.01] and 53% (adjusted *R*^2^ = 0.49) of the variance in GSI [*F*_(12,154)_ = 14.22, *p* < 0.01]. At Step 1 and Step 3, sex was unrelated to EWB, RWB, and RSWB. At Step 2, it was related only to EWB (β = -0.17, *p* < 0.01). At Step 2, only CARE was positively related to EWB (β = 0.20, *p* < 0.01), RWB (β = 0.33, *p* < 0.001) and RSWB (β = 0.30, *p* < 0.001). ANGER was negatively related to EWB (β = -0.27, *p* < 0.001) and RSWB (β = -0.21, *p* < 0.01) while SEEKING and PLAY were only related to EWB (SEEKING: β = 0.21, *p* < 0.01; PLAY: β = 0.24, *p* < 0.01). SADNESS and FEAR were unrelated to all dimensions of spirituality.

At Step 3, among the primary emotions, only the relation of ANGER to EWB remained significant (β = -0.21, *p* < 0.01). Among the personality dimensions, only Agreeableness was positively related to EWB (β = 0.25, *p* < 0.001), RWB (β = 0.31, *p* < 0.01) and RSWB (β = 0.31, *p* < 0.001). Extraversion was positively related to EWB (β = 0.32, *p* < 0.001) and RSWB (β = 0.28, *p* < 0.001) and Conscientiousness was related only to EWB (β = 0.15, *p* < 0.01). Neuroticism and Openness were unrelated to all dimensions of spirituality. Accordingly, all three models showed a significant increase in *R*^2^ between Step 2 and Step 3 (EWB: Δ*R*^2^ = 0.15, *p* < 0.001; RWB: Δ*R*^2^ = 0.11, *p* < 0.001; RSWB: Δ*R*^2^ = 0.15, *p* < 0.001).

## Discussion

This study was intended to further investigate to what extent primary emotions act as a foundation for personality and higher order emotions such as spirituality. In line with previous research, our analyses showed strong relations between primary emotions and personality factors (Davis et al., 2003; Davis and Panksepp, 2011) as well as between personality factors and spirituality (Saroglou, 2010; Unterrainer et al., 2014). Furthermore, regression analyses support our hypothesis that personality factors at least partly mediate the relation between primary emotions and spirituality. However, personality also seems to have a predictive value for spirituality independent of primary emotions.

While primary emotions are the foundation for personality development in the context of Darwinian “continuity” (Bouchard and Loehlin, 2001; Nettle, 2006; Davis and Panksepp, 2011; Panksepp and Watt, 2011) our results suggest that personality likely represents a combination of the expression of an organism’s genes and the influence of environmental factors (Goldberg, 1990).

Specifically, our results indicate that different personality facets seem to be mainly influenced by selected primary emotions that play an important role in the conceptualization of each personality factor; therefore, the strong relations between Extraversion and PLAY as well as between Agreeableness and CARE underline their respective social/interactive focus while the strong relation between Neuroticism with FEAR and SADNESS underlines its close relationship with increased psychopathology in general as well as affective disorders in particular (McCrae and John, 1992; Lang et al., 2001; Panksepp and Biven, 2012).

Interestingly, social and interactive primary emotions and personality facets also had the strongest relationship with religious/spiritual well-being, as CARE and Agreeableness were strongly linked to RWB while PLAY and Extraversion were strongly linked to EWB. In regression analyses, CARE was the most prominent predictor of RSWB. However, it lost its predictive value once the Big Five personality factors were added to the regression model. At this stage, only Extraversion and Agreeableness significantly predicted RSWB, which indicates their mediating role in the relationship between primary emotions and RSWB. Notably, ANGER was the only one of the primary emotions which acted as a negative predictor of EWB, which explains the strong negative relationship between spirituality and aggression in the literature (Unterrainer et al., 2010). Furthermore, the mediating role of personality might be explained by the fact that pure displays of primary emotions are rather uncommon in everyday human life as cortical top-down regulation holds a tight grip over these “neuro-behavioral-psychological tools for survival” (Montag and Panksepp, 2017, p. 5).

The reason why primary emotions appear to have a much greater predictive value for EWB than for RWB might be explained by studies indicating that religious affiliation, but not spirituality, is primarily a culturally influenced trait (D’Onofrio et al., 1999). However, how environmental influences, e.g., diet, prenatal care or stressful life events, interact with genes in the modulation of both religiousness and spirituality should be a goal in future research. Following the theory that religious activities are far less influenced by genes, they might be seen as additional environmental factors which influence spirituality (Perroud, 2009).

Especially unsurprising is the strong relationship between Agreeableness and RSWB given that this personality facet involves “the more humane aspects of humanity – characteristics such as altruism, nurturance, caring and emotional support at the one end of the dimension, and hostility, indifference to others, self-centeredness, spitefulness and jealousy at the other” (Digman, 1990, p. 442). Extraversion, on the other hand, can be seen as located midway between Warmth and Dominance on the Interpersonal Circumplex (McCrae and Costa, 1989) although some argue that this narrow “sociability” definition (McCrae and John, 1992) might be expanded to include positive emotionality as a core element (Watson and Clark, 1997). Correspondingly, EWB can be defined by facets such as hope for a better future, forgiveness and the experience of sense and meaning while RWB is comprised of facets such as the involvement in religious communities, the belief in a higher power and lower amounts of existential fear (Unterrainer et al., 2011).

Understanding the interactions between primary emotions, personality and RSWB could be of particular importance to clinical research, as these dimensions have been discussed in relation to various psychological disorders, e.g., (Horton et al., 2015; Hiebler-Ragger et al., 2016; Unterrainer et al., 2017). For example, substance use disorders have been linked to a deficiency in personality development (Bernstein et al., 1998; Trull et al., 2000) emotion regulation (Goldstein and Volkow, 2011) as well as low RSWB (Unterrainer et al., 2013). In addition, a lower RSWB was found to be linked to affective symptoms in non-clinical previous research (Hiebler-Ragger et al., 2016). However, the established bio-psycho-social model of health and disease, first proposed by Engel (1977), does not include these aspects of religiosity and spirituality. Therefore, the use of the multidimensional RSWB could also be understood as a potential option to stimulate methods that, although currently only covering the immanent state of health, might also adopt transcendent components in the future (Unterrainer et al., 2014). This might especially influence the implementation of intervention techniques based RSWB.

Furthermore, it should be mentioned that Panksepp and colleagues have developed their own measure for the assessment of spirituality within the framework of affective neuroscience, e.g., (Davis and Panksepp, 2011). This scale has been omitted from other versions of the questionnaire as it “is secondary to the neurobiologically justified core dimensions of the ANPS” (Barrett et al., 2013, p. 829). In this study, we also omitted the spirituality scale since we wanted to pursue the idea of capturing spirituality in a multidimensional manner. This multidimensional concept of RSWB includes facets other than the purely emotional form of spirituality which might explain why it showed stronger relations to other primary emotions and personality facets then Panksepp’s conceptualization (Davis et al., 2003; Davis and Panksepp, 2011). In addition, Panksepp and colleagues excluded the primary emotion LUST from their questionnaire arguing that it had little relevance for human personality and that it could not be measured effectively as frank answers would be doubtful (Davis and Panksepp, 2011). At this point, it is therefore impossible to tell what kind of contribution to personality development and the development of higher emotions might be made by this excluded primary emotion.

The findings of this study should be seen as highly preliminary and we acknowledge a need for the results to be replicated in future work. Especially the composition of our sample – that strongly leans toward young, female and Roman-Catholic participants – impairs the generalizability of our findings and underlines the need for future studies. Furthermore, the inclusion of clinical groups would be of special interest since spirituality has also been considered as an important factor in the treatment of severe psychiatric disorders such as depression or addiction (Davis and Panksepp, 2011). Moreover, longitudinal research designs will be necessary to clarify causal directions from primary emotions to personality development and further on to higher emotions such as spirituality. Furthermore, future studies might profit from the inclusion of other aspects of spirituality and religion: For example, the model of intrinsic vs. extrinsic religiosity – stating that “the extrinsically motivated person uses his religion, whereas the intrinsically motivated lives his religion” (Allport and Ross, 1967, p. 434) – has also been linked to personality in previous research (Unterrainer et al., 2014). In addition, future studies might consider potentially psychopathological aspects of religion and spirituality, e.g., regarding personality disorders (Piedmont, 2009). Lastly, future studies should also investigate the validity of our results for different cultural and religious backgrounds.

## Conclusion

our findings not only support the growing evidence for the formation of personality through primary emotions (for an overview see Montag and Panksepp, 2017). They also suggest a strong link between primary emotions and religious/spiritual well-being that seems to be mediated by personality dimensions. More social and interactive personality facets – such as Agreeableness and Extraversion – and primary emotions – such as CARE and PLAY – seem to be especially closely linked to religious/spiritual well-being. However, they seem to be better suited to predicting existential well-being rather than religious well-being. This is in line with previous research (Piedmont, 1999; MacDonald, 2000; Unterrainer et al., 2010) presenting religiousness and spirituality as independent concepts from, but with different associations to, personality.

## Author Contributions

HU conceptualized the work. HD acquired the data. MH-R and HD analyzed and interpreted the data. MH-R, HD, CV, and JF drafted and revised the article. HU and AF supervised the analysis and interpretation of data as well as the drafting and revising of the article.

## Conflict of Interest Statement

The authors declare that the research was conducted in the absence of any commercial or financial relationships that could be construed as a potential conflict of interest.
